# Berberine Ameliorates Subarachnoid Hemorrhage Injury *via* Induction of Sirtuin 1 and Inhibiting HMGB1/Nf-κB Pathway

**DOI:** 10.3389/fphar.2020.01073

**Published:** 2020-07-10

**Authors:** Xiang-Hua Zhang, Lei Peng, Jing Zhang, Yi-Peng Dong, Cheng-Jun Wang, Cang Liu, Da-Yong Xia, Xiang-Sheng Zhang

**Affiliations:** ^1^ Department of Neurosurgery, Beijing Friendship Hospital, Capital Medical University, Beijing, China; ^2^ Department of Neurosurgery, The First Affiliated Hospital of Wannan Medical College (Yijishan Hospital of Wannan Medical College), Wuhu, China

**Keywords:** subarachnoid hemorrhage, inflammation, berberine, HMGB1, Sirtuin 1

## Abstract

Excessive cerebral inflammation plays a key role in early brain injury (EBI) after subarachnoid hemorrhage (SAH). Berberine, an isoquinoline alkaloid isolated from Chinese herb Coptis chinensis, possesses anti-inflammatory, and neuroprotective effects. Here we evaluated the beneficial effects of berberine against SAH-induced inflammatory response and the subsequent brain injury. Our data showed that berberine treatment significantly inhibited microglia activation and proinflammatory cytokines release. Concomitant with suppressed cerebral inflammation, berberine mitigated the subsequent brain injury as demonstrated by improved neurological behavior, reduced brain edema, and decreased neural apoptosis. Moreover, berberine significantly inhibited high mobile group box 1 (HMGB1)/nuclear factor-κB (Nf-κB)-dependent pathway and enhanced sirtuin 1 (SIRT1) expression after SAH. Treatment with ex527, a selective SIRT1 inhibitor, reversed berberine-induced SIRT1 activation and inhibitory effects on HMGB1/Nf-κB activation. In addition, ex527 pretreatment abated the anti-inflammatory and neuroprotective effects of berberine on SAH. Taken together, these findings suggest that berberine provides beneficial effects against SAH-triggered cerebral inflammation by inhibiting HMGB1/Nf-κB pathway, which may be modulated by SIRT1 activation.

## Introduction

Subarachnoid hemorrhage (SAH) remains a fata neurological emergency with high mortality and morbidity rates (Macdonald and Schweizer, 2017; Chen F. et al., 2018; Li et al., 2019). Excessive neuroinflammation is a key pathogenic cascade that contributes to neuronal injury and cell death after SAH (Fan et al., 2017; Lu et al., 2018; Tao et al., 2019; Zhang et al., 2019b). Therefore, pharmacologically reducing cerebral inflammation might be a promising method for the treatment of SAH.

High mobile group box 1 (HMGB1) plays a critical role in triggering neuroinflammation after SAH (Sun et al., 2014). After SAH insults, extracellular HMGB1 binds to toll-like receptor 4 (TLR4) through medullary differentiation factor 88 (Myd88)-dependent pathway, thereby exacerbating nuclear factor-κB (Nf-κB)-dependent proinflammatory mediator production and immune cell infiltration (Wang et al., 2013; Sun et al., 2014). Accumulating evidence has indicated that inhibition of HMGB1/Nf-κB activation could effectively mitigate brain injury after SAH (Zhang et al., 2016a; Xu et al., 2017; Zhang et al., 2019b). Sirtuin 1 (SIRT1) is a type of histone deacetylase and plays an important role in endogenous neuroprotection after SAH (Zhang et al., 2016b). It has been demonstrated that SIRT1 could modulate a variety of biological functions, including transcription, apoptosis, and inflammation. In addition, SIRT1 plays a key role in regulating HMGB1/Nf-κB activation (Chen F. et al., 2018; Zhang et al., 2019b).

Berberine is an isoquinoline alkaloid isolated from Chinese herb Coptis chinensis. Previous studies have demonstrated that berberine has anti-hyperlipidemic, anti-diabetic, anti-bacterial, and anti-cancer properties (Kong et al., 2020). In the central nervous system (CNS), accumulating evidence indicates that berberine possesses potent neuroprotective and anti-inflammatory effects against various neurological disorders, such as brain ischemia, traumatic brain injury, and Alzheimer’s disease (Zhu et al., 2018; Liu D. Q. et al., 2019). In addition, berberine is a small molecule and can easily penetrate the blood-brain barrier (BBB). Intriguingly, recent studies have reported that berberine is a potent SIRT1 agonist and is able to inhibit HMGB1/Nf-κB pathway in a variety of disease models (Sun et al., 2018; Zhang et al., 2019a; Zhou et al., 2020). However, no study has performed to investigate the potential role of berberine in experimental SAH. With this background, we explored the protective effects of berberine after SAH *in vivo* and discussed the possible underlying mechanisms.

## Materials and Methods

### Animals

All procedures in this study were approved by the Animal Care and Use Committee of Capital Medical University and conformed to the ARRIVE guidelines and the *Guide for the Care and Use of Laboratory Animals* published by the National Institutes of Health. Adult male Sprague-Dawley rats (250–300 g) were obtained from the Animal Center of Nanjing University. Rats were acclimated to a 12-h light/dark cycle with free access to food and water under conditions of controlled temperature and humidity. Investigators were blinded to treatment group during experimental tests and data analysis.

### Animal Model of SAH

A prechiasmatic cistern injection models was performed as described previously (Li et al., 2017; Zhang et al., 2019b). Briefly, rats were anesthetized with avertin (200mg/kg). They were then positioned prone in a stereotactic frame, and a hole was drilled into the skull 7.5 mm anterior to the bregma. A total of 0.35 mL of nonheparinized fresh autologous arterial blood from the femoral artery into the drill hole over 20 s with a syringe pump under aseptic conditions. Sham animals underwent the same procedure but were injected with 0.35 mL of physiologic saline instead of blood. After they recovered from the anesthesia, rats were returned to their cages.

### Study Design

In the first set of experiments, 90 rats (100 rats were used, 10 rats died) were randomly divided into the following groups: sham + vehicle, SAH + vehicle, SAH + 20 mg/kg berberine, SAH + 50mg/kg berberine, and SAH + 100 mg/kg berberine. Rats were killed at 24 and 72 h after SAH. Post-assessments included neurologic scores, rotarod performance, brain water content, Western blot, enzyme-linked immunosorbent assays (ELISA), and histopathologic study.

In the second set of experiments, 72 rats (81 rats were used, 9 rats died) were randomly assigned into sham + vehicle, sham + 50mg/kg berberine, SAH + vehicle, SAH + 50mg/kg berberine group, SAH + ex527 group, and SAH + 50mg/kg berberine + ex527 group. Rats were killed at 24 h after SAH. Post-assessments included neurologic scores, rotarod performance, western blot, ELISA, and histopathologic study.

### Drug Administration

Different doses of berberine (20, 50, and 100 mg/kg) (Santa Cruz Biotechnology, Inc., USA) or an equal volume of vehicle was orally administered by gavage at 2 h after SAH insults and then once daily until euthanasia. The pharmacokinetics of oral berberine showed that berberine reached brain within 0.25 h after dosing, and its level in brain was higher than that in the plasma at 4 h after administration (Tan et al., 2013). Ex527, a SIRT1-specific inhibitor, was diluted in dimethylsulfoxide (DMSO) to a concentration of 1% DMSO. Ex527 (10mg/kg) or vehicle was administered intraperitoneally for 3 days before SAH construction. The doses of berberine and ex527 used in the current study were selected according to previous studies (Liu P. et al., 2019; Yu et al., 2019).

### Behavioral Analysis

Neurologic functions were evaluated with an 18-point scoring system reported by Sugawara et al. (2008). An accelerating rotarod was used to assess motor deficits (Zhang et al., 2019b). Rats were adapted to the rotarod for a period of 3 days before randomization. Then the testing was performed at 1, 2, and 3 days after operation. The rotating speed was gradually increased from 4 to 40 rpm over a 5-min period. The time spent on the rotarod was recorded. Three trials were performed, and the individual times from these trials were averaged.

### Brain Water Content

After neurological function test, brain water content was evaluated after SAH. The brains were separated into cerebrum, cerebellum, and brainstem. Each sample was weighed immediately after removal (wet weight), then dried for 72 h at 80°C and weighed to obtain the dry weight. Brain water content was calculated using the following equation: [(wet weight – dry weight)/wet weight] ×100% (Zhang et al., 2019b).

### Western Blot Analysis

Whole cell protein extraction, cytosolic protein extraction, and nuclear protein extraction were extracted according to previous studies (Pan et al., 2014; Zhang et al., 2016a). Equal protein amounts were separated by polyacrylamide gel electrophoresis and transferred to a polyvinylidene difluoride membrane. The membrane was blocked in 5% skim milk for 2 h at room temperature and then incubated with primary antibodies against caspase-3 (1:400, cat #9661, Cell Signaling), SIRT1 (1:200, cat #SC-15404, Santa Cruz), HMGB1 (1:1000, cat #3935S, Cell Signaling), TLR4 (1:200, cat #sc-30002, Santa Cruz), Myd88 (1:200, cat #sc-11356, Santa Cruz), Nf-κB p65 (1:200, cat #sc-372, Santa Cruz), β-actin (1:3000, cat #AP0060, Bioworld Technology, Minneapolis, MN, USA), and Histone H3 (1:3000, cat #BS7416, Bioworld Technology) overnight at 4°C. The membrane was incubated with horseradish peroxidase (HRP)-conjugated IgG for 2 h at room temperature. Detection was performed by enhanced chemiluminescence solution (Thermo Fisher Scientific, Waltham, MA, USA). Band density was quantified with UN-Scan-It 6.1 software (Silk Scientific Inc., Orem, UT, USA).

### ELISA

Brain samples were collected and centrifuged at 12,000 rpm for 20 min at 4°C. The levels of interleukin-1β (IL-1β), interleukin-6 (IL-6), tumor necrosis factor-α (TNF-α), and intercellular adhesion molecule 1 (ICAM-1) were evaluated with ELISA kits according to the manufacturer’s instructions (R&D Systems, Minneapolis, MN, USA).

### Hematoxylin and Eosin (H&E) Staining

Rats were perfused with normal saline followed by 4% paraformaldehyde for fixation. Brain sections (6 μm) were stained with H&E solution according to standard procedures (Zhang et al., 2019b), and then mounted with permount. Staining was visualized under a light microscope.

### Immunofluorescence Staining and TUNEL Staining

Immunofluorescence staining was performed as previously described (Han et al., 2016; Zhang et al., 2016a). Briefly, brain sections (6 μm) were incubated with primary antibodies against Nf-κB p65 (1:50, Santa Cruz) and Iba-1 (1:50, Santa Cruz) overnight at 4°C, and then with Alexa Fluor 594 (Jackson ImmunoResearch Incorporation, West Grove, PA, USA). TUNEL staining was performed according to the manufacturer’s instructions (Roche Inc., Indianapolis, USA). Fluorescence was visualized under a ZEISS HB050 inverted microscope system.

### Statistical Analysis

All data are presented as mean ± SD. Differences among multiple groups were compared by one-way or two-way analysis of variance (ANOVA) with Bonferroni post hoc test. Statistical significance was inferred at *P* < 0.05.

## Results

### Dose-Response Effects of Berberine on SAH

No animals died in the sham + vehicle or sham + 50 mg/kg berberine group. The mortality rate of the rats was 14.3% (5 of 35) in the SAH + vehicle group; 10.8% (8 of 74) in the SAH + berberine group; 25% (4 of 16) in the SAH + ex527 group; and 14.3% (2 of 14) in the SAH + berberine+ ex527group.

**Figure 1:**
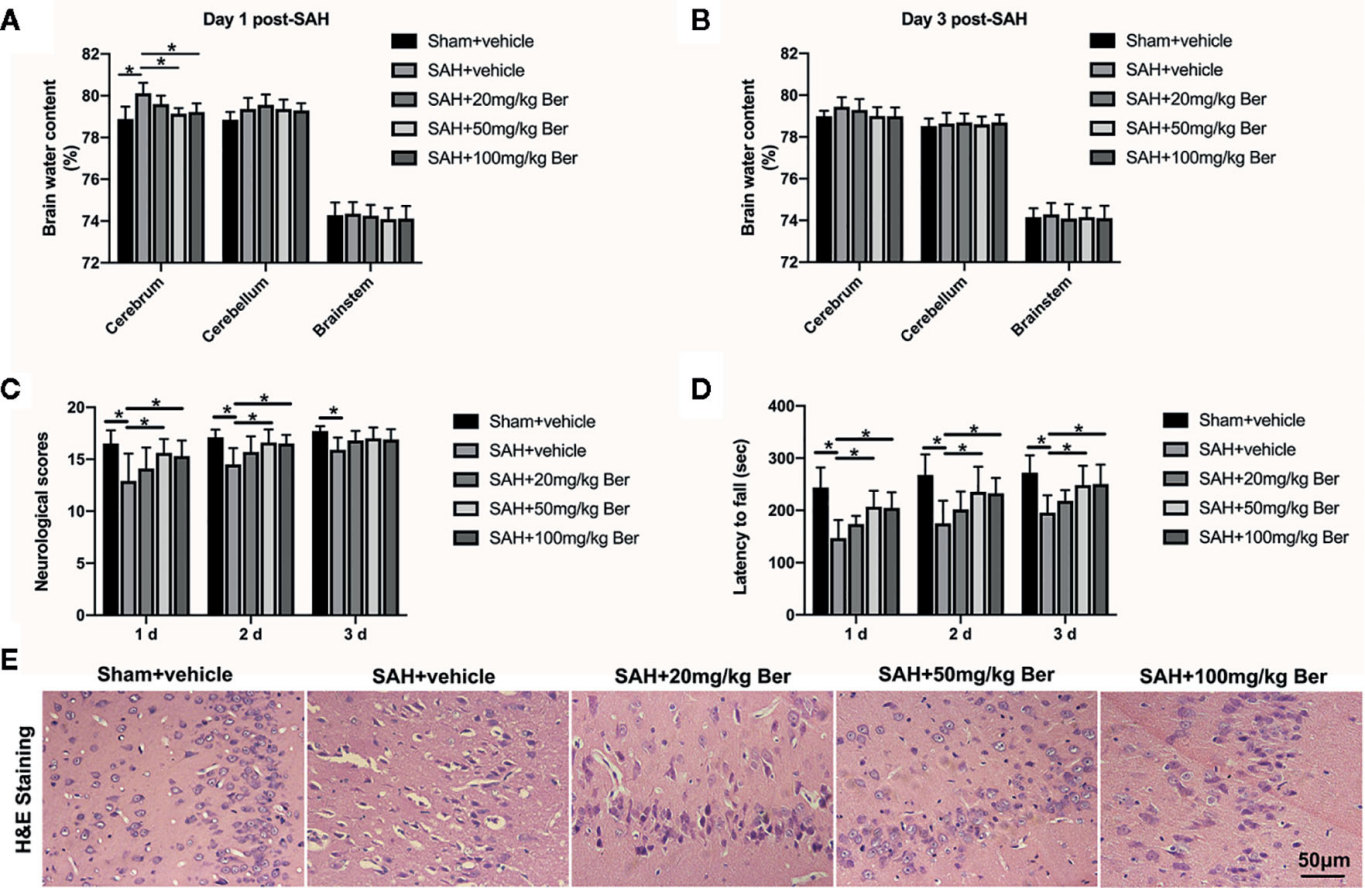
Dose-response effects of berberine on subarachnoid hemorrhage (SAH) in rats. **(A, B)** Effects of three berberine doses on brain water content at 24 h **(A)** and 72 h **(B)** after SAH. n = 6 per group. **(C, D)** Effects of three berberine doses on neurologic scores **(C)** and rotarod performance **(D)** at 24 h, 48 h, and 72 h after SAH. n = 12 per group. **(E)** Representative photomicrographs of H&E staining at 72 h after SAH. n = 6 per group. Bars represent the mean ± SD. ^*^ *P* < 0.05.

The brain water content was measured at 24 and 72 h post SAH. Results showed that berberine at 50 and 100 mg/kg, but not 20 mg/kg, significantly alleviated brain edema at 24 h after SAH (**Figure 1A**). No significant differences in brain edema were detected among the experimental groups at 72 h after SAH (**Figure 1B**). In addition, berberine at 50 mg/kg and 100 mg/kg markedly improved neurologic scores and rotarod performance after SAH. However, 20 mg/kg berberine did not improve neurologic behavior after SAH (**Figures 1C, D**). H*&*E staining further revealed that evident neuronal degeneration characterized by dark-stained nuclei and cytoplasm can be seen in the SAH group. In contrast, berberine treatment could dose-dependently ameliorate neuronal degeneration and improve neural survival (**Figure 1E**).

### Berberine Treatment Reduced Inflammatory Response at 24 h Post-SAH

**Figure 2:**
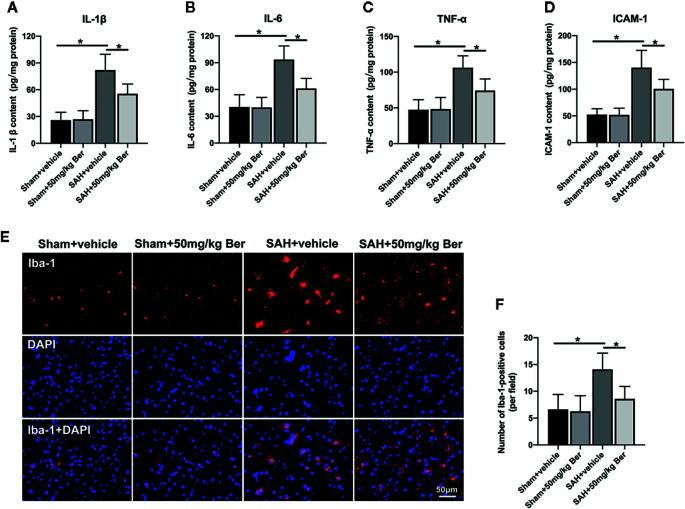
Effects of berberine on inflammatory response after SAH. **(A-D)** Quantification of IL-1β **(A)**, IL-6 **(B)**, TNF-α **(C),** and ICAM-1 **(D)** protein expressions in all groups. n = 6 per group. **(E)** Representative photomicrographs of immunofluorescence staining for Iba-1. **(F)** Quantitative analysis of the Iba-1 immunofluorescence staining. n = 6 per group. Bars represent the mean ± SD. ^*^ *P* < 0.05.

Results showed that SAH significantly induced inflammatory response, as evidenced by increases in IL-1β (**Figure 2A**), IL-6 (**Figure 2B**), TNF-α (**Figure 2C**), and ICAM-1 (**Figure 2D**) release when compared with those of the sham + vehicle group. In contrast, berberine treatment could markedly reduce the enhanced proinflammatory cytokines release after SAH (**Figures 2A–D**). Double-immunofluorescence staining further revealed that SAH induced a significant microglia activation, which could be inhibited by berberine supplementation (**Figures 2E, F**). These data suggest that berberine can effectively inhibit SAH-triggered cerebral inflammation.

### Berberine Treatment Ameliorated Neuronal Apoptosis at 24 h Post-SAH

**Figure 3:**
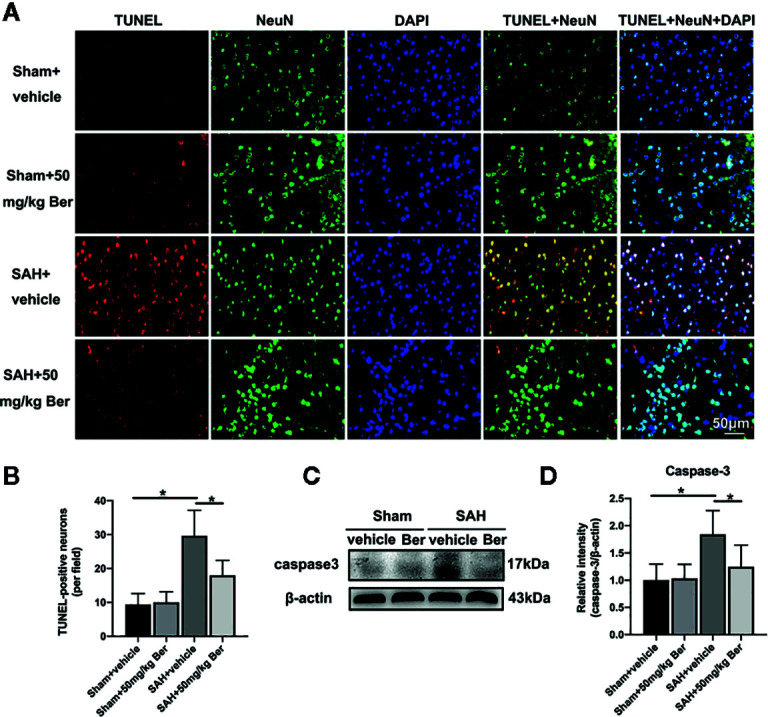
Effects of berberine on neural apoptosis after SAH. **(A, B)** Representative photomicrographs **(A)** and quantification **(B)** of TUNEL staining. n = 6 per group. **(C, D)** Western blot assay **(C)** and quantification for the expression of caspase-3 **(D)** in the indicated groups. n = 6 per group. Bars represent the mean ± SD. ^*^ *P* < 0.05.

As shown, SAH insults exhibited a significant increase in the number of TUNEL-positive neurons as compared with sham + vehicle group, which was markedly decreased after berberine supplementation (**Figures 3A, B**). Western blot data further showed SAH increased caspase-3 expression when compared with sham + vehicle group. In contrast, berberine treatment could evidently inhibit SAH-induced caspase-3 expression (**Figures 3C, D**).

### Berberine Increased SIRT Expression and Inhibited HMGB1/Nf-κB Activation After SAH

**Figure 4:**
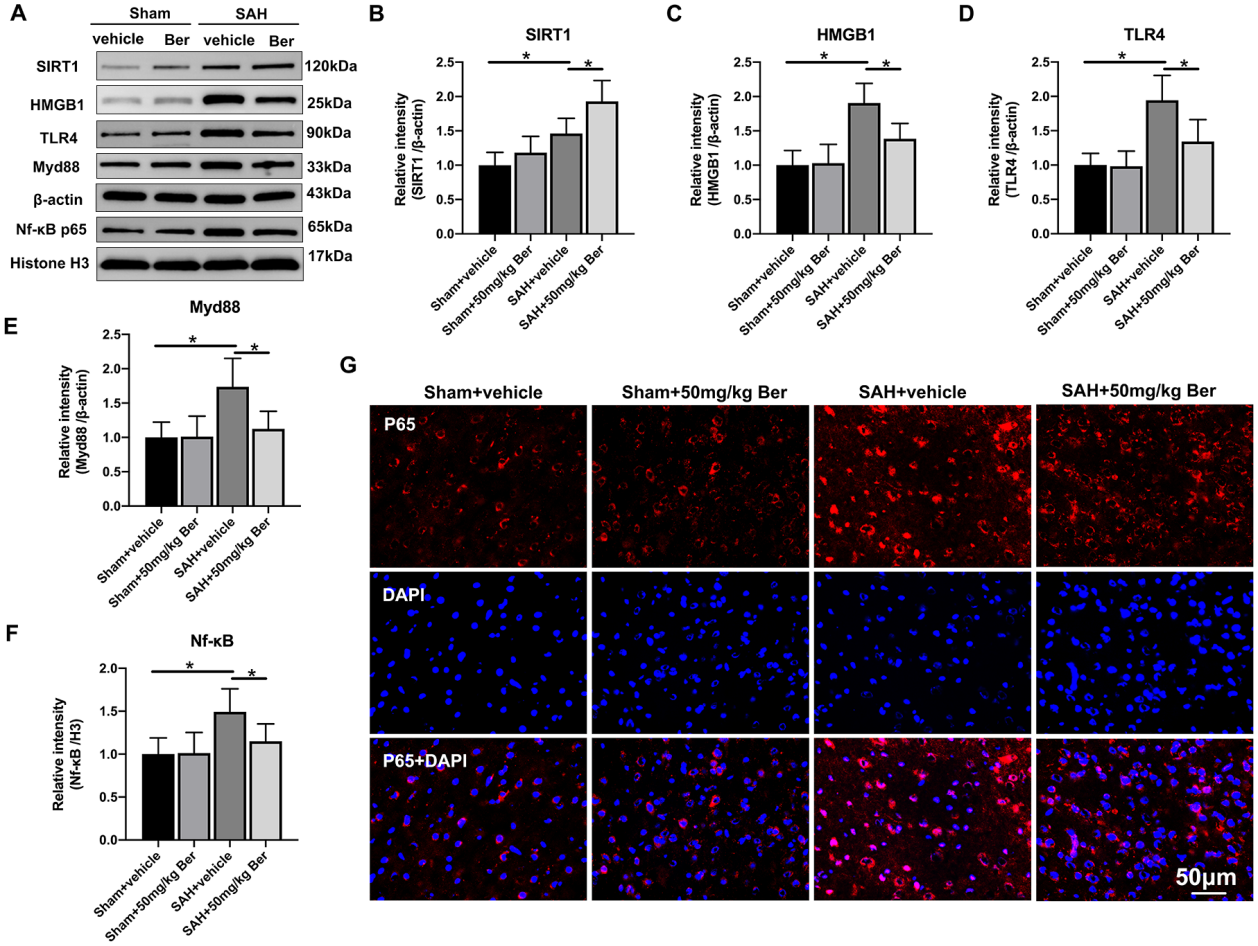
Effects of berberine treatment on SIRT1/HMGB1/TLR4/Myd88/Nf-κB signaling pathway after SAH. **(A–F)** Western blot assay **(A)** and quantification for the expression of SIRT1 **(B)**, HMGB1 **(C),** TLR4 **(D)**, Myd88 **(E)**, and Nf-κB p65 **(F)** in the indicated groups. n = 6 per group. **(G)** Representative photomicrographs of immunofluorescence staining for Nf-κB p65. Bars represent the mean ± SD. ^*^ *P* < 0.05.

Western blot analysis (**Figure 4A**) showed that berberine treatment significantly increased SIRT1 (**Figure 4B**) expression and inhibited the protein levels of HMGB1 (**Figure 4C**), TLR4 (**Figure 4D**), Myd88 (**Figure 4E**), and Nf-κB p65 (**Figure 4F**) after SAH. Double immunofluorescent staining revealed that the expression of Nf-κB p65 was mainly located in the cytoplasm in the sham group. After SAH insults, the Nf-κB p65 nuclear translocation was significantly increased as compared with sham group, which can be evidently suppressed by berberine administration (**Figure 4G**).

### Ex527 Reduced SIRT1 Expression and Enhanced HMGB1/Nf-κB Activation After SAH

**Figure 5:**
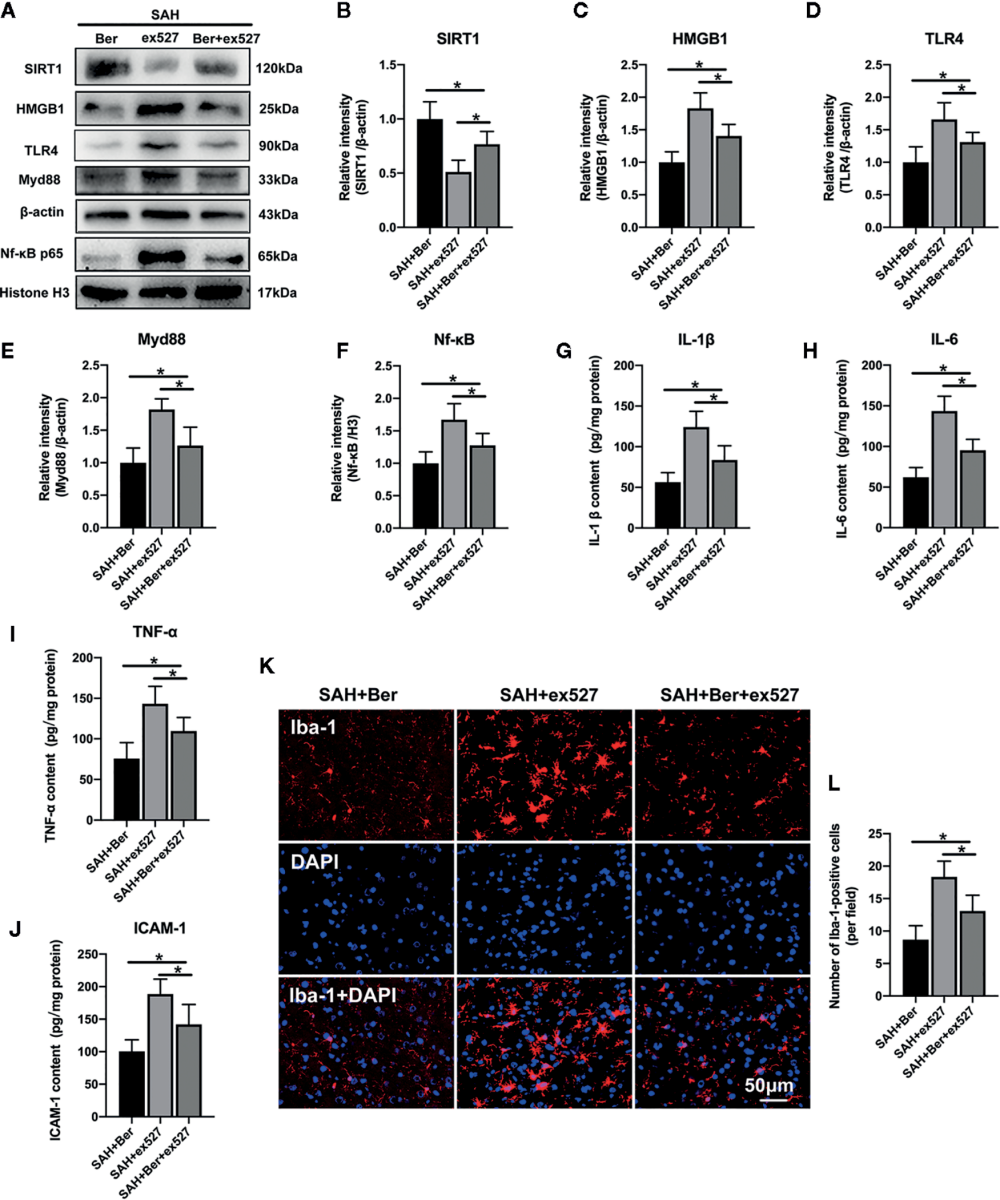
Effects of ex527 treatment on SIRT1/HMGB1/TLR4/Myd88/Nf-κB signaling pathway after SAH. **(A–F)** Western blot assay **(A)** and quantification for the expression of SIRT1 **(B)**, HMGB1 **(C),** TLR4 **(D)**, Myd88 **(E)**, and Nf-κB p65 **(F)** in the indicated groups. n = 6 per group. **(G–J)** Quantification of IL-1β **(G)**, IL-6 **(H)**, TNF-α **(I)** and ICAM-1 **(J)** protein expressions in all groups. n = 6 per group. **(K)** Representative photomicrographs of immunofluorescence staining for Iba-1. **(L)** Quantitative analysis of the Iba-1 immunofluorescence staining. n = 6 per group. Bars represent the mean ± SD. ^*^ *P* < 0.05.

As shown, Western blot analysis (**Figure 5A**) showed that ex527 pretreatment abated berberine-induced SIRT1 expression and the inhibitory effects on HMGB1/Nf-κB activation, as evidenced by decreased SIRT1 expression (**Figure 5B**), and increased protein levels of HMGB1 (**Figure 5C**), TLR4 (**Figure 5D**), Myd88 (**Figure 5E**), and Nf-κB p65 (**Figure 5F**). In addition, ex527 pretreatment reversed the anti-inflammatory effects of berberine after SAH, as demonstrated by increased IL-1β (**Figure 5G**), IL-6 (**Figure 5H**), TNF-α (**Figure 5I**), and ICAM-1 (**Figure 5J**) release, and microglia activation (**Figures 5K, L**).

### Ex527 Reversed the Beneficial Effects of Berberine on Neuronal Apoptosis and Neurological Behavior After SAH

**Figure 6:**
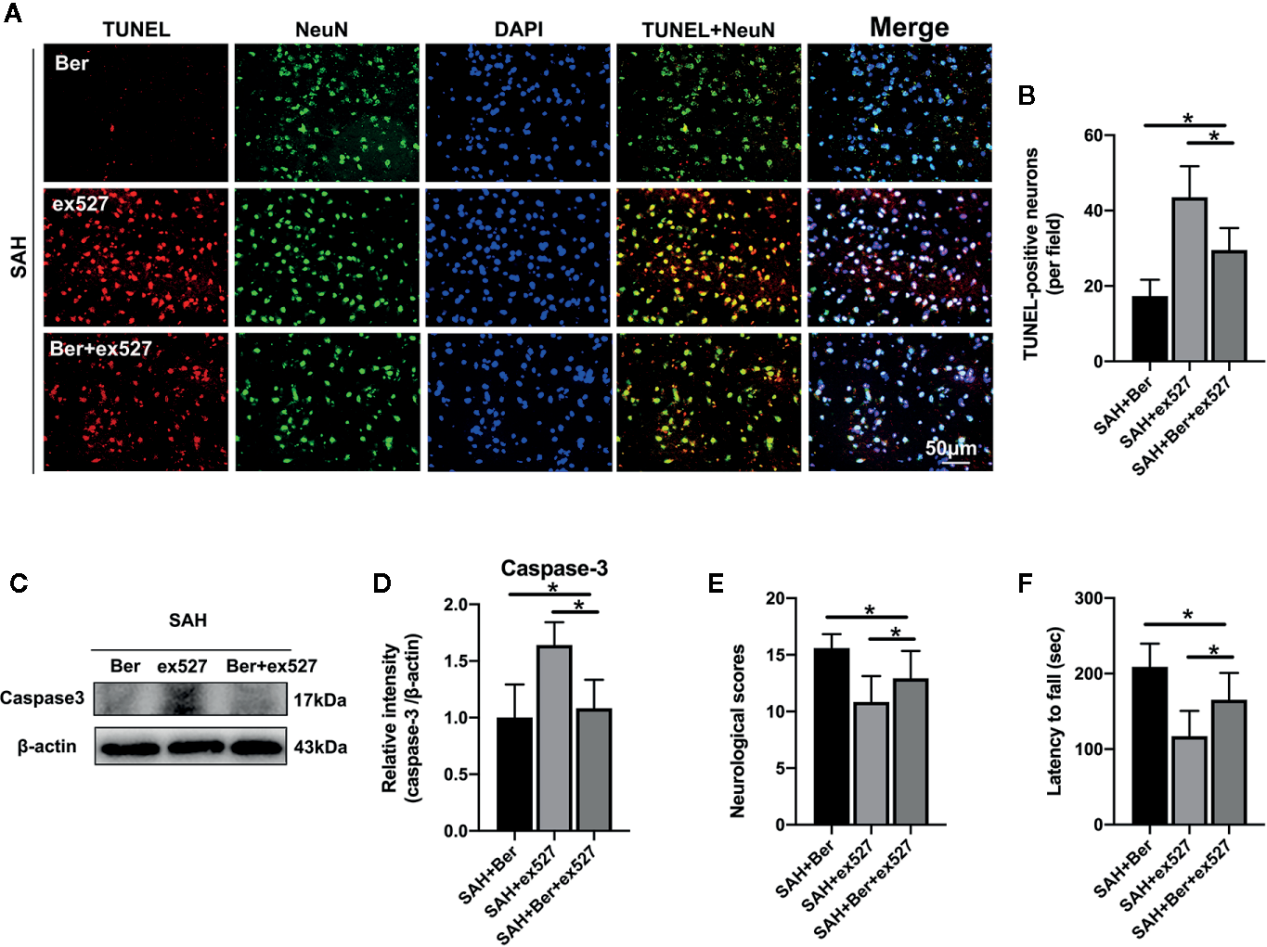
Effects of ex527 on neural apoptosis and neurological behavior after SAH. **(A, B)** Representative photomicrographs **(A)** and quantification **(B)** of TUNEL staining. n = 6 per group. **(C, D)** Western blot assay **(C)** and quantification for the expression of caspase-3 **(D)** in the indicated groups. n = 6 per group. **(E, F)** Effects of ex527 on neurologic scores **(E)** and rotarod performance **(F)** at 24 h after SAH. n = 12 per group. Bars represent the mean ± SD. ^*^ *P* < 0.05.

TUNEL staining revealed that ex527 pretreatment reversed the berberine-induced decrease in the number of TUNEL-positive neurons after SAH (**Figures 6A, B**). Western blot analysis further showed that the reduced caspase-3 expression by berberine was significantly enhanced after SIRT1 inhibition by ex527 (**Figures 6C, D**). In addition, ex527 pretreatment eliminated the berberine-induced improvement in neurological scores and rotarod performance (**Figures 6E, F**).

## Discussion

In the current study, we verified the beneficial effects of berberine in the early period after SAH. The major findings can be summarized as follows: (1) Berberine administration mitigated SAH-induced cerebral inflammation. (2) Berberine provided neuroprotection against early brain injury (EBI) after SAH as demonstrated by improved neurobehavior performance, decreased neural apoptosis, and alleviated brain edema. (3) Berberine supplementation inhibited HMGB1/Nf-κB signaling and enhanced SIRT1 activation after SAH. (4) Ex527, a selective SIRT1 inhibitor, effectively suppressed berberine-induced SIRT1 activation and HMGB1/Nf-κB inhibition, and reversed the beneficial effects of berberine against SAH. These findings provide evidence that berberine mitigates EBI after SAH primarily through increasing SIRT1 and inhibiting HMGB1/Nf-κB signaling.

Mounting evidence has shown that cerebral inflammation induced by activation of microglia is a vital component of the pathological cascade in EBI after SAH (Liu et al., 2018; Lu et al., 2018; Tao et al., 2019). After SAH, resident microglia and peripheral macrophages migrate to the site of injury and release excessive inflammatory cytokines to further damage neurons (Sun et al., 2014; Li et al., 2017). Inhibition of neuroinflammation has been proved to effectively reduce EBI and improve long-term prognosis after SAH (Li et al., 2015; Tao et al., 2019). Consistent with previous researches (Lu et al., 2018; Tao et al., 2019), we found that activation of microglia and expression of inflammatory cytokines were markedly increased after SAH, which were associated with the aggravated brain edema, neural apoptosis, and neurological impairment.

Berberine is a well-known natural compound that possess a variety of biological functions. In the CNS, studies have shown that berberine could easily pass the BBB and provide beneficial effects against a variety of neurological disorders (Liu D. Q. et al., 2019; Zhang et al., 2019a). But no study has yet investigated the potential effects in experimental SAH. It has been reported that berberine has a potent anti-inflammatory function and is able to reduce cerebral inflammation in a series of acute brain injuries (Seo et al., 2018; Zhu et al., 2018). Consistent with previous reports in other research fields (Seo et al., 2018; Zhu et al., 2018), we revealed that berberine administration could effectively inhibit microglia activation and the subsequent pro-inflammatory cytokines release. Concomitant with the decreased cerebral inflammation, berberine significantly improved neurological behavior, reduced brain edema, and alleviated neural apoptosis. These suggest that berberine could ameliorate EBI by inhibition of inflammatory response after SAH. However, the potential molecular mechanisms of berberine’s anti-inflammatory effects remain unclear.

Activation of the HMGB1/Nf-κB pathway plays an important role in cerebral inflammation after SAH (Sun et al., 2014; Chaudhry et al., 2018). HMGB1 has been shown to be widely distributed in the nucleus of nearly all eukaryotic cells, including brain cells. After SAH insults, HMGB1 triggers inflammatory response by interaction with toll-like receptors, such as TLR4. TLR4 then triggers a signaling pathway that leads to the activation of Nf-κB and the subsequent induction of proinflammatory cytokines and chemokines (Sun et al., 2014; Okada et al., 2019). Growing evidence has shown that inhibition of HMGB1/Nf-κB could mitigate cerebral inflammation and improve neurological outcomes after SAH (Sun et al., 2014; Zhang et al., 2019b). Consistent with previous reports, our study revealed that HMGB1/Nf-κB pathway was significantly activated and was associated with the robust cerebral inflammation after SAH. Additionally, our data showed that berberine markedly inhibited HMGB1/Nf-κB activation after SAH. Actually, numerous studies have demonstrated that berberine could inhibit HMGB1/Nf-κB pathway in a variety of diseases models, such as brain ischemia, endotoxic shock, and acetaminophen hepatotoxicity (Li et al., 2016; Lee et al., 2017; Zhu et al., 2018). These observations suggest that HMGB1/Nf-κB pathway plays a key role in cerebral inflammation and berberine could inhibit HMGB1/Nf-κB pathway-mediated inflammatory response and the subsequent brain injury after SAH.

How berberine modulates HMGB1/Nf-κB pathway remains unknown. A possible mechanism might involve SIRT1. SIRT1, a member of nicotinamide adenine dinucleotide (NAD^+^)-dependent protein deacetylases, could regulate a variety of cellular functions, such as immune response, apoptosis, and metabolism by deacetylating its target proteins (Herskovits and Guarente, 2014; Zhang et al., 2016b). It has been demonstrated that HMGB1/Nf-κB activation is closely related to HMGB1 acetylation (Chen X. et al., 2018; Zhang et al., 2019b). Accumulating evidence has shown that SIRT1 modulates HMGB1 hyperacetylation and inhibits HMGB1 translocation release in different research fields (Hwang et al., 2015; Chen X. et al., 2018). Intriguingly, recent studies in other research areas has proved that berberine is a potent SIRT1 activator (Sun et al., 2018; Zhang et al., 2019a). In agreement with previous studies, our results showed that berberine could significantly upregulate SIRT1 expression and inhibit HMGB1/Nf-κB activation to reduce brain injury after SAH. In contrast, the SIRT1-selevtive inhibitor ex527 abated the upregulation of SIRT1 and the inhibitory effects on HMGB1/Nf-κB activation by berberine, thereby aggravated SAH-induced inflammatory response and brain injury. Together with our experimental results, these observations suggest that berberine supplementation inhibits HMGB1/Nf-κB activation-mediated inflammatory response by enhancing SIRT1 expression after SAH.

In the current study, we chose male rats as the test subjects. Although more women than men suffer from SAH in clinic, the overall mortality and neurological outcomes are not better in males despite their younger age (Hamdan et al., 2014). In addition, experimental studies have showed that male rats experience a greater bleed, greater severity of EBI, and reduced survival as compared to age-matched female rats (Friedrich et al., 2013). Whether berberine could protect female rats from SAH remains unclear. A previous study has demonstrated that estrogen replacement therapy could reduce cerebral aneurysmal rupture by alleviating the vascular inflammation (Maekawa et al., 2017). We therefore speculate that berberine might still have protective effects in female rats after SAH by reducing cerebral inflammation. But additional studies are needed to unravel this novel area. To data, numerous clinical trials have explored the physiological relevance of berberine in human subjects and showed that berberine might prevent obesity, improve insulin resistance, and decrease hyperlipidemia (Yin et al., 2008; Xu et al., 2019). However, the toxicity of berberine might cause altered liver function, gastric troubles, hematotoxicity, and damage to immune cells (Singh and Sharma, 2018). Thus, more work is required to make these preclinical studies more translatable to the clinic.

There are several limitations in our study. Firstly, we are not sure that other functions and signaling pathways contribute to the neuroprotective effects of berberine on EBI after SAH. In addition to regulate SIRT1 and HMGB1/Nf-κB signaling, berberine can target nuclear factor-erythroid 2-related factor 2, nod-like receptor pyrin domain-containing 3 inflammasome, and adenosine monophosphate-activated protein kinase, which also has been involved in the pathophysiology of EBI after SAH (Zhou et al., 2017; Yerra et al., 2018). Secondly, microglia polarization plays an important in cerebral inflammation after SAH (Lu et al., 2018; Tao et al., 2019). In the current study, we demonstrated that berberine could inhibit microglia activation after SAH, but we did not characterize the exact microglia phenotype. Thirdly, whether berberine treatment has a wide therapeutic time window and exerts long-lasting neuroprotective effects after SAH remain unknown. Lastly, although previous reports have investigated the pharmacokinetics of oral berberine in rodents (Liu et al., 2010; Tan et al., 2013), we did not evaluate the systemic berberine exposure in the present study. Considering that the current research is a pilot study, future work is still needed to decipher these important questions.

## Conclusion

In summary, we provide the first evidence that berberine could mitigate SAH-induced cerebral inflammation by inhibiting HMGB1/Nf-κB pathway, which may be modulated by SIRT1activation. Although more research is required, our results indicate that berberine might be a promising drug candidate for treatment of SAH.

## Data Availability Statement

The datasets generated for this study are available on request to the corresponding authors.

## Author Contributions

X-HZ and X-SZ performed the studies and wrote the manuscript. LP and JZ participated in creating the experimental animal model. Y-PD, C-JW, and D-YX contributed to the Western blotting and the immunohistochemical and immunofluorescence staining. CL, D-YX, and X-SZ contributed to the design and analysis of the study and revised the manuscript. All authors contributed to the article and approved the submitted version.

## Funding

This work was supported by grants from Natural Science Research Project in Higher Education of Anhui Province (No. KJ2018A0253 for D-YX) and the Science Research Project of Professional personnel of the First Affiliated Hospital of Wannan Medical College (No. YR201911).

## Correction note

﻿A correction has been made to this article. Details can be found at: 10.3389/fphar.2025.1540873.

## Conflict of Interest

The authors declare that the research was conducted in the absence of any commercial or financial relationships that could be construed as a potential conflict of interest.

## Abbreviations

BBB, blood-brain barrier; CNS, central nervous system; DMSO, dimethylsulfoxide; EBI, early brain injury; ELISA, enzyme-linked immunosorbent assays; H&E, hematoxylin and eosin; HMGB1, high mobile group box 1; HRP, horseradish peroxidase; ICAM-1, intercellular adhesion molecule 1; IL-1β, interleukin-1β; IL-6, interleukin-6; Myd88, medullary differentiation factor 88; NAD^+^, nicotinamide adenine dinucleotide; Nf-κB, nuclear factor-κB; SAH, subarachnoid hemorrhage; SIRT1, sirtuin 1; TLR4, toll-like receptor 4; TNF-α, tumor necrosis factor-α.
