# Correction: Berberine ameliorates subarachnoid hemorrhage injury via induction of Sirtuin 1 and inhibiting HMGB1/Nf-κB pathway

**DOI:** 10.3389/fphar.2025.1540873

**Published:** 2025-07-14

**Authors:** Xiang-Hua Zhang, Lei Peng, Jing Zhang, Yi-Peng Dong, Cheng-Jun Wang, Cang Liu, Da-Yong Xia, Xiang-Sheng Zhang

**Affiliations:** ^1^ Department of Neurosurgery, Beijing Friendship Hospital, Capital Medical University, Beijing, China; ^2^ Department of Neurosurgery, The First Affiliated Hospital of Wannan Medical College (Yijishan Hospital of Wannan Medical College), Wuhu, China

**Keywords:** subarachnoid hemorrhage, inflammation, berberine, HMGB1, Sirtuin 1

There was a mistake in Figure 4A as published, concerning the NF-kB p65 protein band. It appears that the protein band was improperly cropped during the figure preparation. Furthermore, the Western blot image included in Figure 4A does not align with the complete Western blot image. The corrected Figure 4 appears below.

**FIGURE 4:**
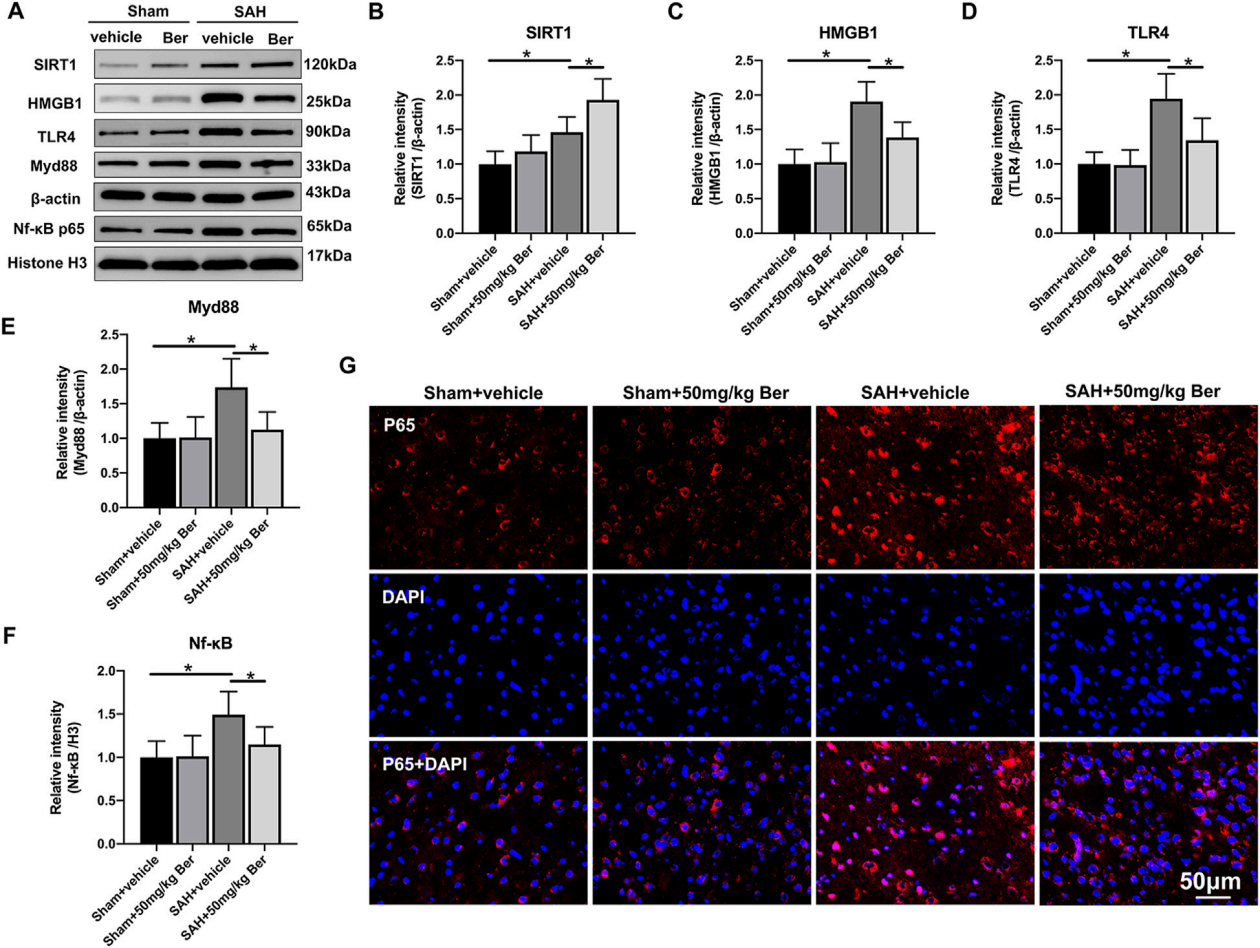
Effects of berberine treatment on SIRT1/HMGB1/TLR4/Myd88/Nf-κB signaling pathway after SAH. **(A–F)** Western blot assay **(A)** and quantification for the expression of SIRT1 **(B)**, HMGB1 **(C)**, TLR4 **(D)**, Myd88 **(E)**, and Nf-κB p65 **(F)** in the indicated groups. n = 6 per group. **(G)** Representative photomicrographs of immunofluorescence staining for Nf-κB p65. Bars represent the mean ± SD. ^*^ *P* < 0.05.

The original version of this article has been updated.

